# Bilingualism Enhances Reported Perspective Taking in Men, but Not in Women

**DOI:** 10.3389/fpsyg.2021.679524

**Published:** 2021-05-17

**Authors:** Samaneh Tarighat, Andrea Krott

**Affiliations:** ^1^Faculty of Foreign Languages, Islamic Azad University Tehran North Branch, Tehran, Iran; ^2^School of Psychology, University of Birmingham, Birmingham, United Kingdom

**Keywords:** perspective taking, gender differences, bilingualism, bilingual advantage, Theory of Mind

## Abstract

Bilingual speakers have often been found to be superior in taking the perspective of another person. Also, females are commonly found to have enhanced perspective taking (PT) abilities compared with males, with male PT being generally more easily affected by external factors. The present study investigated whether bilingualism improves PT in males more strongly than in females. In total, 108 bilingual and 108 matched monolingual adults, with equal numbers of males and females, filled in the PT subscale of the Interpersonal Reactivity index. While monolinguals showed the typical result of females scoring higher on PT than males, scores of male and female bilinguals did not differ, with both bilingual groups scoring as high as female monolinguals. Thus, bilingualism enhanced self-reported PT only in males, suggesting that male PT can be enhanced through socialization.

## Introduction

Being bilingual or multilingual is undoubtfully important for social, political, and economic reasons. More controversial is that it comes with cognitive, linguistic, and sociocommunicative advantages (see review by Poarch and Krott, 2019). For instance, bilinguals are reported to have significantly better executive functioning such as working memory, mental switching, and inhibition abilities (e.g., Lehtonen et al., 2018). They also possess higher metalinguistic awareness (Galambos and Goldin-Meadow, 1990; Bialystok and Barac, 2012) and are better at prioritizing and multitasking (Poarch and Bialytsok, 2015). Another proposed advantage is superior perspective taking (PT) skills, that is, the skill or propensity of taking the perspective of another person (e.g., Bialystok and Senman, 2004; Farhadian et al., 2010; Rubio-Fernández and Glucksberg, 2012; Greenberg et al., 2013; Fan et al., 2015; Javor, 2016; Liberman et al., 2017; see review by Schroeder, 2018). However, PT has also been found to be affected by gender, with females having superior PT than males (Charman et al., 2002; Kessler et al., 2014; Van der Graaff et al., 2014; Chopik et al., 2017; see review in Christov-Moore et al., 2014). Male PT seems generally less automatic and more susceptible to external factors than female PT (Brehm et al., 1984; Theodoridou et al., 2013; Tremblay et al., 2020). It is, therefore, possible that bilingualism improves PT in males more strongly than in females.

## Perspective Taking

The present study is concerned with PT, the ability to grasp the thoughts, beliefs, and visual experience of another person. PT is used in the literature interchangeably with the terms Theory of Mind (ToM), cognitive empathy, and mentalizing, that is, the ability to understand the mental state of oneself or others (e.g., Zaki and Ochsner, 2012; Christov-Moore et al., 2014). While PT is a more common term in studies that rely on self-report measures, ToM is a term often adopted in experimental studies.

Perspective taking is very closely related to the concept of empathy, “the reactions of one individual to the observed experiences of another” (Davis, 1983, p. 113). While some research treat the two concepts as two different, but closely connected, constructs (e.g., Barnes-Holmes et al., 2004; Harwood and Farrar, 2006), others understand empathy as an umbrella term to PT. For instance, Davis (1980) sees PT as one of four components of empathy, next to fantasy, empathic concern, and personal distress, and Zaki and Ochsner (2012) distinguish three facets of empathy: PT (= mentalizing or cognitive empathy), experience sharing (= affective empathy), and prosocial concern. In the present study, we will investigate PT, thus cognitive empathy and mentalizing, leaving other aspects of empathy aside.

Stronger PT abilities are generally associated with positive features. For instance, higher PT skills are related to better social functioning and higher self-esteem (Davis, 1983). PT skills also predict the size of a person’s social network (Stille and Dunbar, 2007), and children with higher PT scores are more popular among their peers (Slaughter et al., 2015). Furthermore, better PT can help the development of prosocial behavior (Schroeder, 2018), and individuals’ propensity toward PT is related to altruistic behavior (Tusche et al., 2016). On a societal level, individuals with better PT skills more easily suppress automatic expressions of racial bias, contributing to a reduced intergroup bias (Todd et al., 2011). Similarly, PT is a personality trait that reflects one’s attitudes and possibly openness toward diversity (Hemer et al., 2019). It has therefore been argued that developing PT in citizens is crucial for cultivating a civic identity (Johnson, 2015) and building a diverse democratic society (Reason, 2011).

While strong PT skills are generally associated with positive features, there is a “dark” side, which is seldom discussed. Schlegel (2020) explored the downsides of strongly perceiving others’ emotions and distinguished two aspects: an intrapersonal danger and an interpersonal danger. The intrapersonal danger arises because PT may negatively impact wellbeing when experiencing negative life events or when being overly obsessed with the suffering of others. The interpersonal danger arises because PT may be used to hurt or manipulate others. Ding et al. (2015) reported more cases of dishonesty among children who were trained in ToM. Furthermore, Tarrant et al. (2012) pointed out that in cases where an individual highly identifies with other members in the same group, higher PT can lead to more stereotyping of outsiders. Such a condition can lead to the formation of negative judgments about out-group members and damage intergroup relations. Thus, while on the whole, higher PT has been associated with many positive outcomes, there might be a certain level of PT that is beneficial, with either very high or very low levels of PT having negative effects.

### Bilingualism and Perspective Taking

PT has been investigated in bilingual participants along with its neighboring constructs ToM and spatial PT, as well as with empathy as a more general construct. A bilingual advantage has been reported with a range of methods.

The most frequently used methods are ToM tasks. These often take the form of false-belief tasks, in which participants need to distinguish between their own knowledge and that of another person. Children only slowly develop ToM, typically failing ToM tests before age 4. In a meta-analysis of 16 studies (comprising of, in total, 655 monolingual and 628 bilingual children), Schroeder (2018) reported a small to medium-sized ToM advantage in bilingual compared with monolingual children, depending on the analysis used. The small-size effect is comparable with the effect of early education interventions on cognitive, school, and social outcomes (Schroeder, 2018). While a bilingual ToM advantage has mainly been studied in children, it has also been found for adults (Rubio-Fernández and Glucksberg, 2012; Javor, 2016).

Bilingual ToM studies have been conducted in various countries and, thus, across various cultures and languages, suggesting that the bilingual ToM advantage is universal. For instance, Bialystok and Senman (2004) found an advantage in a Canadian population of bilingual preschoolers with various language backgrounds compared with monolingual children, while Farhadian et al. (2010) found a bilingual advantage for Kurdish–Persian bilingual preschoolers compared with Persian monolingual preschoolers in Iran.

A related type of PT to ToM is visual–spatial PT. Just like ToM, it involves the separation and management of two (or more) representations. Bilinguals have shown evidence of superior abilities in this aspect of PT as well. For example, Greenberg et al. (2013) reported an advantage in visual–spatial PT in bilingual compared with monolingual children. Bilingual children were better at calculating the correct view of an observer on an array of blocks. Similarly, bilingual children were superior at seeing an alternative interpretation of ambiguous figures, which involves the inhibition of the prevalent interpretation (Bialystok and Shapero, 2005; Wimmer and Marx, 2014). However, such findings are not without exceptions. For instance, Ryskin et al. (2014) studied adult listeners’ ability to accommodate the spatial PT of others in conversation and found no bilingual advantage. In fact, in some cases, adult bilinguals showed more difficulty in taking the spatial perspective of a communication partner.

Bilingual PT skills have also been studied in argumentative essays. Hsin and Snow (2017) reported superior PT skills, namely, perspective acknowledgment and perspective articulation, in primary school bilinguals with a language-minority background compared with English-speaking monolinguals in the United States. These superior skills were particularly remarkable since the bilingual children were less proficient in the language of the test (English).

Finally, a bilingual PT advantage has also been found in self-reports. Javor (2016) compared monolingual and bilingual adults on the Interpersonal Reactivity Index (IRI) developed by Davis (1980). Bilinguals scored higher than monolinguals on all subscales of the questionnaire (i.e., PT, fantasy scale, empathetic concern, and personal distress). Important for the present study is the PT subscale, which assessed the tendency to spontaneously adopt the psychological point of view of others (e.g., “I try to look at everybody’s side of a disagreement before I make a decision”). The same study found a bilingual advantage also with a more implicit measure, that is, a ToM measure. Importantly, the PT scores on the IRI were correlated with the ToM performance. This suggests not only that bilinguals show superior PT in experimental and self-report measures but also that both types of measures tap into the same cognitive processes.

Several reasons have been suggested for the reported bilingual superiority of PT. Schroeder (2018) distinguished between three accounts: a sociopragmatic account (Goetz, 2003; Kovács, 2009; Bialystok et al., 2012; Fan et al., 2015), an executive functioning account (Goetz, 2003; Bialystok and Senman, 2004; Kovács, 2009; Greenberg et al., 2013; Javor, 2016), and a metalinguistic awareness account (Goetz, 2003; Diaz and Farrar, 2018). The sociopragmatic account rests on the observation that bilinguals share either one or both of their languages with another person. Bilinguals, therefore, have a very good awareness that other people might have different mental states than themselves and are used to taking on another person’s mental state (Kovács, 2009; Bialystok et al., 2012; Rubio-Fernández and Glucksberg, 2012). Heightened sociopragmatic awareness might also be caused by the fact that bilingualism is strongly related to biculturalism. Being bicultural means to be confronted with two cultural systems of rules and beliefs. Bilinguals might, thus, be more aware of the fact that one and the same situation can be viewed from different perspectives and that other people might not share the same perspective as them. This account is supported by the finding that children from large families who regularly encounter individuals who differ from one another in their behavior and their beliefs show a higher level of PT (Selcuk et al., 2018). It is also congruent with the finding that multiracial students show no significant growth in PT over the years spent in college in contrast to single-race students (Hemer et al., 2019).

The executive functioning account of PT rests on findings that bilinguals are superior in general cognitive skills (Carlson and Meltzoff, 2008; Bialystok and Viswanathan, 2009) and that executive functioning is involved in ToM performance (Devine and Hughes, 2014). In addition, children’s performance on a verbal working memory task has been shown to mediate the relationship between bilingualism and performance on a false-belief task (Nguyen and Astington, 2014). The superior executive functioning skills are argued to develop due to the demand of a bilingual to control their two languages, which is assumed to involve the suppression of one language in order to use the other one. These executive function skills might help bilinguals with PT in that they could help suppress one’s own mental states and focus more on the mental states of others.

Finally, the metalinguistic account explains bilingual PT advantages in terms of bilingual superior metalinguistic skills (Galambos and Goldin-Meadow, 1990; Bialystok and Barac, 2012; Diaz and Farrar, 2018). Importantly, it has been shown that metalinguistic skills are related to ToM development (Doherty, 2000), and language learning can improve ToM development (Pyers and Senghas, 2009).

It is not clear yet which account of the bilingual PT advantage is the correct one or whether the advantage might stem from a combination of the accounts. The present study does not try to address this question. However, given the accounts for gender differences in PT presented in the next section, it is important to note that all accounts emerge from the special social (or cultural) situation that bilinguals find themselves in.

### Gender Differences in Perspective Taking

It has been well established that females exhibit higher PT and, more generally, higher empathy, than males (see review by Christov-Moore et al., 2014). Differences have been reported with experimental tasks and self-report measures, particularly in children and adolescents. For instance, girls have been found to outperform their male counterparts on false-belief tasks (Charman et al., 2002; Selcuk et al., 2018), and 5- to 13-year-old girls were more skilled than boys at guessing the cause of an infant’s distress (Catherine and Schonert-Reichl, 2011). Furthermore, in a population of 10- to 13-year-olds, girls were better than boys at identifying the feelings and intentions of characters in a story (Bosacki and Astington, 1999).

Gender differences seem to increase with age and time. For instance, Van der Graaff et al. (2014) reported higher PT levels among 497 female Dutch adolescents compared with males using a self-report measure over 6 years, from age 13 to 18. Both genders increased in PT, but girls scored increasingly higher than boys over time. Similarly, Hemer et al. (2019) measured PT with the Personal and Social Responsibility Inventory in a sample of well over 10,000 American college students. They reported that, with each year of higher education, female PT scores increased more strongly than male scores.

Gender differences have not only been found in children and adolescents, but also in adults. Chopik et al. (2017) investigated empathy with all its subcomponents in a sample of 104,365 participants from 63 different countries, using the IRI (Davis, 1983). Females reported higher PT as well as higher empathic concern and total empathy compared with males.

Males seem not to take the perspective of others as automatically as females. Brehm et al. (1984) asked children how much money they would donate to another child in need. Boys increased the rate of donations when being asked to imagine themselves in the other child’s circumstances, while girls would give similar donations with and without being prompted to take the other child’s perspective. Similarly, male adult PT seems more malleable than female PT. Tremblay et al. (2020) found that adults’ empathy was increased when asked to empathize with people showing facial expressions of pain, but more so for men.

The reasons for a gender difference in PT and empathy are not clear. Some scholars believe that they are, to a large degree, due to biological differences and are, therefore, heritable (see a comprehensive review in Christov-Moore et al., 2014). It is assumed that evolutionary changes occurred in order for females to detect and react to newborns’ signals (Adenzato et al., 2017). In contrast, others imply that gender differences are primarily a result of social stereotypes and cultural beliefs about gender roles in society and are, therefore, developed (e.g., Baron-Cohen et al., 2005; Chakrabarti and Baron-Cohen, 2013). Gender differences could also arise due to a combination of both.

Arguments for a biological cause of gender differences in PT and empathy stem from studies with non-human animals and human infants as well as studies on the effects of hormones on empathy. In their comprehensive review of gender differences in empathy, Christov-Moore et al. (2014) described a female empathy dominance in the animal kingdom. For instance, female baboons show stronger and more specific matching of yawns than male baboons (Palagi et al., 2009). Also, female rodents show greater sensitivity to the pain of unfamiliar rodents than male rodents (Langford et al., 2006), and female chimpanzee bystanders show more consolatory behavior to distressed individuals than males (Romero et al., 2010), which suggests that the female advantage in empathy exists not only in humans but also in other species and is, therefore, biological. However, these findings could be a result of the fact that only species with similar female social roles have been studied, i.e., species with females as the primary caretakers. Thus, gender differences in empathy could simply root in a common evolutionary history of female roles in maternal care (Babchuk et al., 1985).

Another argument in favor of a biological basis of gender differences in empathy is that human female infants exhibit rudimentary forms of empathy more strongly than male infants, responding more strongly to social emotional stimuli than male infants. For instance, female neonates cry more often and for longer when hearing another infant cry (e.g., Simner, 1971), they make more eye contact (Hittelman and Dickes, 1979), and more likely to orient to faces (Connellan et al., 2000). While these findings do not necessarily mean that females have a genetic predisposition to be more empathetic or to take someone else’s perspective, these behavioral differences show that female infants are more socially interested and, therefore, have more opportunities to learn about other people’s states and perspectives. This, in turn, might mean that they can more easily learn to take someone else’s perspective (see also Christov-Moore et al., 2014).

A further argument for a biological account of gender differences in empathy are effects of hormones on empathy. For instance, administration of testosterone, the main sex hormone in men, decreases the ability to empathize (van Honk et al., 2011). When female participants received a single dose of sublingual testosterone, it significantly decreased their ability to infer other people’s mental states from the eye region of their faces. In addition, men performed comparably with women in PT in a computerized PT task once intranasal oxytocin, a hormone related to social bonding, reproduction, and childbirth, had been administered (Theodoridou et al., 2013). Since the same study did not find an effect of oxytocin in a self-report measure of empathic reactions to a scenario, implicit measures seem to be more sensitive to oxytocin than self-report measures.

Contrary to biological arguments, gender differences in empathy have also been argued to be rooted in culture and socialization (Baron-Cohen et al., 2005). A major cultural stereotype held in many societies is that women are more empathetic, kinder, and more soft-hearted than men. In contrast, many male heroes are portrayed as more aggressive and less empathetic. In accordance with these gender stereotypes, men may feel the need to act less empathically in order to appear more masculine. Similarly, women might have the desire to comply with social norms or exaggerate their empathetic side, especially when self-report measures are used (Wager and Ochsner, 2005). In line with the argument that PT is affected by expectations, PT scores can be affected by the circumstances of a task. For instance, accuracy in judging interpersonal behavior can be affected by what participants believed is measured. Females have been found to score lower when they believed a test measured judgment skills in the military (a typical male skill), while males scored lower when they believed the test was about judgment skills important for social workers (a typical female skill; Horgan and Smith, 2006). Thus, females show the tendency to try harder to take other people’s perspectives in a task if they believe that what is measured is related to a stereotypical female role. Importantly, females’ desire to comply with cultural and social norms does not mean that they show higher PT scores only on self-report measures. With practice, enhanced PT might become second nature to them without the active desire to conform to stereotypes. As a consequence, females might take another person’s PT more automatically and easily, which would be reflected not only in self-report measures but also in more implicit PT tasks.

## The Present Study

Given the evidence that PT in males is more malleable than in females, the present study investigated whether bilingualism enhances PT in males more strongly than in females. We tested PT in Iran, where an estimated 33.7 million bilinguals live, as 42% of the population reported to be bilingual in a 2003 census (Hamidi, 2005). A sum of 14 different language varieties are currently spoken in Iran (CIA Factbook, 2010), while the official language taught in schools and broadcast over the country through terrestrial and satellite channels is Persian. This makes it possible for those who speak other languages at home to become familiar with the dominant language, leading to a large number of balanced bilinguals. As a result, the Iranian population lends itself very well to a bilingualism study.

We measured PT with the PT subscale of the IRI developed by Davis (1980). As mentioned, the PT subscale assesses the tendency to spontaneously adopt the psychological point of view of others. We did not assess the other subscales of the IRI because we were only interested in bilingual PT, not in bilingual fantasy, empathic concern, or personal distress. The IRI has been widely employed in research and in different countries around the globe ever since its development (e.g., Alterman et al., 2003; Fernández et al., 2011; Gilet et al., 2013). In addition and as mentioned previously, bilingual adults score higher on the PT subscale of the IRI than monolinguals (Javor, 2016). While it is a self-report measure, it correlated with the study of Javor with the Theory of Mind PT measure, supporting the notion that both measures of PT tap into very similar cognitive processes. Furthermore, females score higher than males on the PT subscale of the IRI (Chopik et al., 2017). It is therefore a good measure to study a potential interaction of bilingualism and gender.

As outlined above, bilingualism has been shown to increase PT skills and the propensity of PT. While there are various potential reasons for this advantage (sociopragmatic, executive functioning, and metalinguistic skills, see above), all of them are developmental accounts in that changes are believed to arise from sociological and cultural differences between bilingual and monolingual speakers. Therefore, if gender differences in PT were purely biological in nature, then bilingualism should not affect them. Yet, if gender differences in PT are at least partly affected by sociological and cultural experience, then bilingualism might increase PT in the two genders differently. Additionally, it seems that PT in males is more susceptible to environmental and biological factors. As we have seen, for males, PT is less automatic (Brehm et al., 1984; Theodoridou et al., 2013; Tremblay et al., 2020). Therefore, bilingualism might raise PT more strongly in males. This would mean that the PT difference between male and female bilinguals should be smaller than that between male and female monolinguals.

## Materials and Methods

### Participants

We calculated that we would need 51 participants in each participant group to achieve a medium effect size of 0.5 and a power of 0.7 (α = 0.05, two-tailed, independent *t*-test). Participants were recruited using the snowball method, starting with acquaintances of the first author^1^. Each monolingual participant was recruited to match as much as possible a bilingual participant in terms of age, gender, and education level.

In order to confirm that participants were either monolingual or bilingual, we asked them to fill in a background questionnaire, which contained questions about gender, age, education, and various questions about their language background. They were asked to indicate the number of languages they spoke and had learned throughout their lives, their proficiency in each language, the daily percentage of language use, the age of acquisition (AoA) of each language, and the years they spent learning that language or living in an environment where the language was spoken. For each language, participants had to choose their level of proficiency on a scale from 0 to 10, being instructed that 0 means “I know less than five words in this language” and 10 means “I have native-like proficiency in this language and I speak, listen, read, and write it like my mother tongue and I don’t have a foreign accent.” This led to 216 participants, distributed evenly over the four groups of male and female monolinguals and bilinguals, all living in Iran (mostly Tehran).

Table 1 lists the group characteristics^1^. The two groups showed clear differences in terms of their language proficiency and usage. Importantly, bilinguals rated their proficiency as at least 7 out of 10 for both languages, but the large majority rated it as 9 or 10, meaning that almost all bilingual participants were balanced bilinguals. Monolinguals rated any L2 proficiency as 4 or less. Monolinguals spoke Persian, while bilinguals spoke Persian and a variety of other languages, namely Azeri (42), Kurdish (30), Armenian (18), English (10), Lori (3), Lak (2), Turkmen (2), and Mazandarani (1). Persian was the mother tongue only for a small number of the bilingual participants, namely, the English speakers. All other participants were born into language minority groups, meaning that they spoke a different language than Persian at home and were exposed to Persian in society, at school, and through the media. The Persian–English bilinguals were children of former Iranian immigrants or long-term visitors to English speaking countries who had now returned to their native country.

**TABLE 1:** Mean values of participant characteristics (SDs in parentheses).

	Bilinguals		Monolinguals	
	Female (*N* = 54)	Male (*N* = 54)	Female (*N* = 54)	Male (*N* = 54)
Age (in years)	34.61 (11.91)	31.48 (12.43)	34.46 (12.79)	31.57 (12.89)
Education (1–5)	2.93 (1.11)	2.44 (1.21)	2.90 (1.05)	2.47 (1.09)
L1 proficiency (0–10)	9.96 (0.20)	9.84 (0.42)	10.00 (0.00)	9.88 (0.42)
Daily% use of L1	52.92 (28.17)	56.84 (29.19)	92.83 (20.82)	98.87 (2.41)
Years of L1 exposure/usage	29.35 (11.78)	28.54 (13.73)	34.22 (13.53)	31.01 (11.33)
L2 proficiency (0–10)	9.16 (0.99)	9.15 (0.99)	1.67 (1.61)	1.37 (1.66)
Daily% use of L2	51.00 (24.96)	54.11 (28.57)	1.91 (1.74)	9.88 (0.42)
L2 AoA	5.25 (4.03)	5.02 (4.15)	12.40 (7.10)	11.27 (6.98)
Years of L2 exposure/usage	25.87 (13.28)	25.24 (14.82)	3.45 (5.55)	2.25 (3.12)

Since age and education of participants might affect PT scores, we checked group differences on both variables using either an analysis of variance (for age), with gender (male vs. female) and bilingualism (bilingual vs. monolingual) as fixed factors plus a gender x bilingualism interaction, or a Wilcoxon rank sum test (for education), testing group-differences pairwise. The four participant groups were matched in age: there were no main effects of gender [*F*(1,212) = 3.1, *p* = 0.088] or bilingualism [*F*(1,212) = 0, *p* = 0.987] on age and no gender by bilingualism interaction [*F*(1,212) = 0, *p* = 0.944]. All participants had at least a high school diploma. However, while the groups of bilingual and monolingual participants were matched in terms of education level (females: *W* = 1,411, *p* = 0.769; males: *W* = 1,467.5, *p* = 0.955), females in both language groups had a higher education level than males (monolinguals: *W* = 1,822, *p* = 0.022; bilinguals: *W* = 1,822.5, *p* = 0.022). We therefore checked whether education level correlated with PT scores (see section “Results”). If so, it could potentially explain any gender differences.

### Assessment of Perspective Taking

We measured PT with a Persian translation of the PT section of the IRI by Davis (1983) (see Appendix A). This section consists of seven statements about spontaneously taking the perspective of others (e.g., “I try to look at everybody’s side of a disagreement before I make a decision”; for the full list of items, see Appendix A). Participants indicate how well the statements describe them by choosing the appropriate letter on a scale from A (“Does not describe me well”) to E (“Describes me very well”). Five of the questions are scored in order (*A* = 0 to *E* = 4), while two questions are scored in reverse (*A* = 4 to *E* = 0). The total score for a participant is the sum of the scores for the seven items and can vary between 0 and 28.

## Results

**FIGURE 1:**
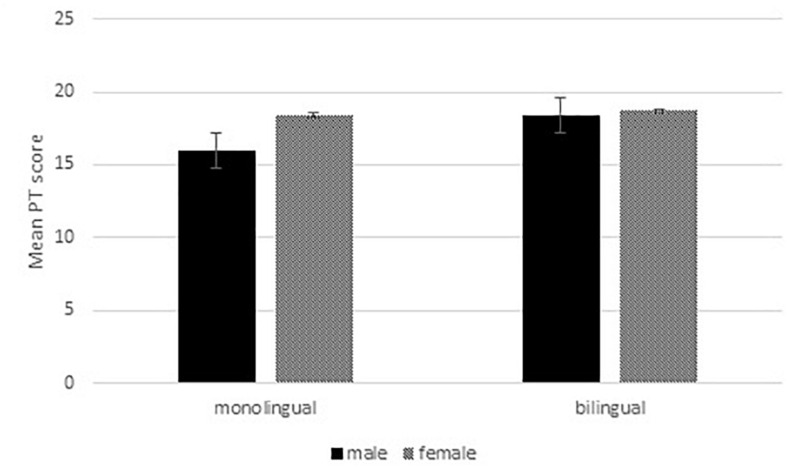
Effect of gender (male vs. female) and bilingualism (bilingual vs. monolingual) on PT scores (range 0–28). Error bars represent standard errors.

Figure 1 shows the PT scores for the four participant groups. Since females had a higher education level than males, we checked whether education level correlated with PT scores, but this was not the case [Pearson’s *r*(214) = 0.06, *p* = 0.345)] We therefore did not take education level into account for any analysis of PT. To assess the effects of gender and bilingualism on PT, we conducted a 2 (gender: male vs. female) × 2 (bilingualism: bilingual vs. monolingual) analysis of variance with PT scores as a dependent variable. We found significant main effects of gender [*F*(1,212) = 5.8, *p* = 0.017) and bilingualism [*F*(1,212) = 6.5, *p* = 0.012) on PT scores, as well as gender by bilingualism interaction [*F*(1,212) = 4.0, *p* = 0.048). *Post hoc* tests show that for monolingual participants, females had higher PT scores than males [*t*(105.9) = 3.0, *p* = 0.004]. For bilingual participants, there was no significant difference between males and females [*t*(102.7) = 0.3, *p* = 0.755]. Furthermore, female monolingual participants had similar PT scores to female bilingual participants [*t*(105.9) = −0.4, *p* = 0.705]. However, male monolingual participants had lower PT scores than male bilingual participants [*t*(100.8) = −3.1, *p* = 0.002]. In other words, monolingual male participants had lower PT scores than the other groups, who all scored very similarly.

## Discussion

The results of our study showed that bilingualism did not affect PT in both genders equally. While monolinguals showed the expected pattern of females scoring higher on PT than males (e.g., Christov-Moore et al., 2014), there was no difference between genders for bilingual participants. Both bilingual females and males scored very similarly to monolingual females. In other words, bilingualism raised PT only for men.

Our results are generally in line with previous findings on bilingual superiority in PT and ToM (e.g., Bialystok and Senman, 2004; Farhadian et al., 2010; Rubio-Fernández and Glucksberg, 2012; Greenberg et al., 2013; Fan et al., 2015; Liberman et al., 2017; Schroeder, 2018), especially with the study by Javor (2016), who tested PT with the same questionnaire as the present study. However, the bilingual PT advantage in our study was only observed for men, not for women. Our findings bring forth the idea that the bilingual advantage reported in other PT studies might have been driven exclusively or primarily by a male advantage.

The results add to the evidence that male PT is less automatic and more malleable than female PT. Previous studies had shown that male PT increases more strongly than that of females when being asked to take the perspective of others (Brehm et al., 1984; Tremblay et al., 2020), and only males engage more strongly in PT when given the female hormone oxytocin (Theodoridou et al., 2013). The present results show another way to increase automaticity of PT in males, namely, through life experiences (i.e., bilingualism). Furthermore, in contrast to monolingual males, bilingual males reported the same level of PT as both monolingual and bilingual females. Thus, while bilingualism seems to increase the automatization of PT in males, it raises PT only to a certain level. This could be a consequence of the dark side of high PT, with extreme levels of PT leading to stress (Schroeder, 2018), dishonesty (Ding et al., 2015), and the tendency to manipulate or hurt others (Schlegel, 2020) as well as to stereotype others (Tarrant et al., 2012). Our results, thus, suggest that there might be an optimal level of PT. Note that the lack of an effect of bilingualism on female PT cannot be due to a ceiling effect. With scores of 18.4 for monolingual females and 18.7 for bilingual females and a possible maximum score of 28, there is plenty of room for higher scores.

A consideration for the lack of an effect of bilingualism on females in the present study is that participants stemmed from Iran, a collectivist culture. Individuals of collectivist cultures have been reported to have enhanced PT compared with individuals of individualist cultures (Wu and Keysar, 2007; Wu et al., 2013; Kessler et al., 2014; Chopik et al., 2017). The monolingual PT baseline in our sample might, therefore, have been relatively high. Monolinguals in individualist cultures might score lower, meaning that in such cultures, female PT could potentially be raised by bilingualism as well.

The bilingual PT advantage has been explained in terms of enhanced executive functioning, higher metalinguistic awareness, and/or better sociopragmatic skills (Schroeder, 2018). Our results raise the question whether bilingualism enhances any of these skills primarily in males. For instance, females have been reported to have superior inhibition and self-regulation skills (Yuan et al., 2008). Bilingualism might raise these skills in males to a female level. Alternatively, bilingual males, in contrast to monolingual males, might have sociopragmatic skills at the level of females. Bilingual males might have a heightened awareness of other individuals’ mental states compared with their monolingual counterparts. This could be rooted in bilinguals’ constant awareness of the language(s) communication partners speak. It could also be a result of their biculturalism, that is, their experience with different cultural norms and rules (see section “Introduction”).

Gender differences in PT have been proposed to be due to biological sex differences or driven by socialization (e.g., Baron-Cohen et al., 2005; Christov-Moore et al., 2014). Since bilinguals differ from monolinguals in terms of socialization, the present findings highlight the role of socialization and culture for PT. If the female PT advantage was only biological in nature, bilingualism should not be able to alter PT levels and cause the disappearance of the gender differences in PT. Alternatively, if gender differences in PT are partly rooted in biological differences, then our results show that socialization can overrule such differences. If, however, PT gender differences are purely a result of socialization, then our results suggest that bilingualism can override cultural influences of masculine and feminine stereotypes that demand males to appear stronger and females to be empathic. As reviewed above, bilingualism might soften the gender differences in PT by, for instance, raising executive function skills or sociopragmatic skills in males.

It needs to be noted that the present study used a self-report measure of PT. Therefore, mediating factors might come into play, such as lack or existence of self-confidence, underestimating or overestimating ones’ true performance in reality when imagining a given situation, and social values and beliefs (Healey and Grossman, 2018). In order to overcome such drawbacks, more implicit measures such as false-belief tasks (the unexpected transfer test or the unexpected-contents test) or other ToM tasks could be used. However, despite the possibility of self-report measures being a stronger reflection of cultural stereotypes and beliefs, bilingualism and gender advantages have been found with both self-report and ToM tests (but see visuospatial tasks such as those in Mohr et al., 2010; Ryskin et al., 2014). Also, Javor (2016) found a correlation between the responses on the IRI and a ToM task. We therefore would expect to find results comparable with the present ones when employing a more implicit task like a ToM task. However, since such tasks might be less affected by developmental factors and socialization than self-reports, it might be that bilingualism would only reduce, but not completely erase, gender differences in such tasks. Such a finding would be interesting from a sociological point of view. It would mean that gender difference in bilingual PT would be partly social/cultural and partly cognitive.

To conclude, we found a bilingual advantage only for male participants, not for females, suggesting that male PT can be made more automatic through the bilingual experience. It remains to be seen whether more implicit measures of PT as well as individualist populations might show a bilingualism effect also in females. Besides, our findings speak to gender differences in PT. They support the notion that gender differences in PT are, at least partly, rooted in socialization and culture.

## Data Availability Statement

The dataset presented in this study can be found at https://osf.io/rmgnw/?view_only=9ddc6316c3d64dad8bf615e38cee8a43.

## Ethics Statement

Ethical review and approval was not required for the study on human participants in accordance with the local legislation and institutional requirements. Written informed consent for participation was not required for this study in accordance with the national legislation and the institutional requirements.

## Author Contributions

ST designed the study and collected the data. AK conducted the statistical analysis. ST and AK co-wrote the article. Both authors contributed to the article and approved the submitted version.

## Conflict of Interest

The authors declare that the research was conducted in the absence of any commercial or financial relationships that could be construed as a potential conflict of interest.

## Footnotes

1 Preliminary results of a subset of the current data have been published in a study by Rashtchi et al. (2020).
