# Supplementary material for: Bilingualism Enhances Reported Perspective Taking in Men, but Not in Women
Source: Front Psychol. 2021 May 17;12:679524. doi: 10.3389/fpsyg.2021.679524 (PMC8165194; doi:10.3389/fpsyg.2021.679524)
Supplement: Supplementary file 1 [file Data_Sheet_1.docx]

Supplementary Material

# Appendix A

Perspective Taking Scale of the Interpersonal Reactivity Index^[[1]](#footnote-1)^ (IRI) (Davies, 1980) and its Persian Translation

| Read each of the following statements and rate how well each of them describes you. Please check the box that corresponds to the number which applies to you for each item: | | Does not describe me well |  |  |  | Describes me well |
| --- | --- | --- | --- | --- | --- | --- |
| IRI8 | Before criticizing somebody, I try to imagine how I would feel if I were in their place. | A | B | C | D | E |
| IRI9 | If I'm sure I'm right about something, I don't waste much time listening to other people's arguments. | A | B | C | D | E |
| IRI10 | I sometimes try to understand my friends better by imagining how things look from their perspective. | A | B | C | D | E |
| IRI11 | I believe that there are two sides to every question and try to look at them both. | A | B | C | D | E |
| IRI12 | I sometimes find it difficult to see things from the "other guy's" point of view. | A | B | C | D | E |
| IRI13 | I try to look at everybody's side of a disagreement before I make a decision. | A | B | C | D | E |
| IRI14 | When I'm upset at someone, I usually try to "put myself in his shoes" for a while. | A | B | C | D | E |

**پرسشنامه اتخاذ دیدگاه**

جملات زیر تا چه حدی شما را به درستی توصیف می کنند؟ در جدول علامت بزنید. (الف: این جمله من را اصلا به درستی توصیف نمی کند؛ ه: این جمله من را کاملا به درستی توصیف می کند.)

| ه | د | ج | ب | الف | قبل از انتقاد از کسی، سعی می کنم تصور کنم اگر من جای او بودم چه حسی داشتم. | 27 |
| --- | --- | --- | --- | --- | --- | --- |
| ه | د | ج | ب | الف | اگر مطمئن باشم که در مورد مسئله ای درست فکر می کنم، برای گوش دادن به عقاید دیگران در این باره وقت تلف نمی کنم. | 28 |
| ه | د | ج | ب | الف | گاهی سعی می کنم با تصور مسایل از دیدگاه دوستانم، آنها را بهتر درک کنم. | 29 |
| ه | د | ج | ب | الف | من معتقدم هر مسئله ای دو وجه دارد و سعی می کنم به هر دو وجه آن توجه کنم. | 30 |
| ه | د | ج | ب | الف | گاهی برای من مشکل است که از دیدگاه طرف مقابل به مسایل نگاه کنم. | 31 |
| ه | د | ج | ب | الف | من سعی می کنم قبل از اینکه تصمیمی بگیرم دیدگاه های همه طرف های موافق و مخالف را در نظر بگیرم. | 32 |
| ه | د | ج | ب | الف | وقتی از کسی ناراحتم، سعی می کنم برای چند لحظه خودم را جای او بگذارم. | 33 |

1. Scoring: Scores are added up, but items IRI9 and IRI12 are reverse scored. [↑](#footnote-ref-1)
